# Sex-Specific Effects of Respiratory Muscle Endurance Training on Cycling Time Trial Performance in Normoxia and Hypoxia

**DOI:** 10.3389/fphys.2021.700620

**Published:** 2021-08-06

**Authors:** Julie Chambault, Grégorine Grand, Bengt Kayser

**Affiliations:** Institute of Sport Sciences, University of Lausanne, Lausanne, Switzerland

**Keywords:** hypoxia, altitude, endurance, respiratory muscle training, sex, cycling, time trial

## Abstract

**Objectives:** We tested the hypotheses that respiratory muscle endurance training (RMET) improves endurance cycling performance differently in women and men and more so in hypoxia than in normoxia.

**Design:** A prospective pre–post cross-over study with two testing conditions.

**Methods:** Healthy and active women (seven, 24 ± 4 years, mean ± standard deviation [SD]) and men (seven, 27 ± 5 years) performed incremental cycling to determine maximum oxygen consumption (VO_2peak_) and power output (W_peak_) and on different days two 10-km cycling time trials (TTs) in normoxia and normobaric hypoxia (FiO_2_, 0.135, ~3,500 m equivalent), in a balanced randomized order. Next they performed supervised RMET in normoxia (4 weeks, 5 days/week, 30 min/day eucapnic hyperpnea at ~60% predicted maximum voluntary ventilation) followed by identical post-tests. During TTs, heart rate, ear oximetry reading, and W_peak_ were recorded.

**Results:** The VO_2peak_ and W_peak_ values were unchanged after RMET. The TT was improved by 7 ± 6% (*p* < 0.001) in normoxia and 16 ± 6% (*p* < 0.001) in hypoxia. The difference between normoxic and hypoxic TT was smaller after RMET as compared with that before RMET (14% *vs*. 21%, respectively, *p* < 0.001). All effects were greater in women (*p* < 0.001). The RMET did not change the heart rate or ear oximetry reading during TTs.

**Conclusion:** We found a greater effect of RMET on cycling TT performance in women than in men, an effect more pronounced in hypoxia. These findings are congruent with the contention of a more pronounced performance-limiting role of the respiratory system during endurance exercise in hypoxia compared with normoxia and more so in women whose respiratory system is undersized compared with that of men.

## Introduction

In conditions of normoxia, the capacity of the cardiorespiratory oxygen transport is the main limiting factor for maximum oxygen consumption (VO_2max_) (Bassett and Howley, 2000; Lundby et al., 2017). Quantified with incremental testing protocols to exhaustion, VO_2max_ cannot be maintained for long. For aerobic endurance-type exercises such as running or cycling, it is the highest maintainable fraction of VO_2max_ for a given time that sets the best performance for a given distance such as the marathon, with a multitude of other interacting factors contributing to ceiling effects such as the energy cost of locomotion, development of neuromuscular fatigue, availability of energy substrate, and thermoregulation (Jones et al., 2020). In conditions of normoxia, the respiratory system is thought not to be an important limiting factor for maximum aerobic capacity in young, healthy, but not especially trained men, who possess excess respiratory system capacity (Dempsey et al., 2020). However, the respiratory system can be a limiting factor for aerobic capacity in highly trained endurance athletes, such as cross-country skiers, who have very high cardiac outputs and may experience a limitation in their expiratory flow because their high aerobic capacity leads to an “excessive” ventilatory demand outstripping their ventilatory system capacity (McKenzie, 2012; Dempsey et al., 2020) or in master athletes due to aging lungs (Burtscher et al., 2011). For endurance type of effort, fatigue of the respiratory muscle is also currently thought to be a limiting factor (Boutellier, 1998; Spengler and Boutellier, 2000; Dempsey et al., 2020).

An exposure to hypoxia leads to a ventilatory response, which increases ventilation for a given oxygen uptake. With increasing degrees of hypoxia, the respiratory system thus progressively becomes a limiting factor for cardiorespiratory oxygen transport and hence aerobic performance (Ferretti and Prampero, 1995). At extreme altitudes, the increase in oxygen uptake resulting from a further increase in ventilation is even thought to be offset by the very oxygen cost of that increase in ventilation, making the extra effort futile (Cibella et al., 1999). Apart from this limitation to oxygen uptake imposed by ventilation in conditions of low tension of inspiratory oxygen, the increase in ventilation in hypoxia for a given oxygen uptake also increases the work of breathing, thereby placing more stress on the respiratory muscles for a given level of metabolic activity of the whole organism.

For so much of exercise physiology (Ansdell et al., 2020), the form and function of the respiratory system during exercise have been studied mostly in men. In recent years, authors such as Sheel et al. (2016) began studying women and found that there are sex-based differences in the anatomy and physiology of the human respiratory system. It is currently understood that women are more susceptible to limitations in the respiratory system during exercise than men (Molgat-Seon et al., 2018; Dempsey et al., 2020). This difference is likely to be exacerbated in hypoxia. Archiza et al. (2020) compared diaphragm fatiguability from inspiratory loading in men and women and found that while men and women fatigued to the same extent in normoxia, diaphragm fatigue was greater in women compared with that in men in acute hypoxia.

In normoxia, respiratory muscle training (RMT) delays diaphragm fatigue and can improve the performance of endurance exercise (Boutellier, 1998; Spengler and Boutellier, 2000; Sheel, 2002; Illi et al., 2012; HajGhanbari et al., 2013; Segizbaeva et al., 2014; Shei, 2018). RMT can consist of inspiratory or combined inspiratory/expiratory muscle strength training (i.e., respiratory muscle strength training [RMST]) or of respiratory muscle endurance training (RMET, by means of sustained normocapnic hyperpnea) (Illi et al., 2012). In normoxia, it was reported that the metaboreflex solicited for a given level of diaphragm fatigue may be less pronounced in women compared with men (Geary et al., 2019). For exercise in hypoxia, a recent review (Álvarez-Herms et al., 2019) concluded that RMT reduced the fatigue of the respiratory muscles, delayed the metaboreflex activation of the respiratory muscles, and consequently allowed for better maintenance of SaO_2_ and blood flow to the active locomotor muscles. No studies to date have reported a direct comparison of the effects of RMT in women *vs*. men on endurance performance in normoxia and hypoxia. If the effects would be greater in women as compared with those in men, this would be of relevance for training programs. Therefore, we tested the hypotheses that RMET improves the performance of endurance exercise both in normoxia and in hypoxia but more so in hypoxia and more so in women than in men.

## Methods

The study protocol was approved by the Geneva University Hospitals Research Ethics Committee and was conducted in accordance with the latest Declaration of Helsinki; all participants signed an informed consent form. Fourteen healthy recreationally active participants (seven men, 27 ± 5 years, 154 ± 47 min exercise/week; seven women, 24 ± 4 years, 80 ± 30 min exercise/week; [means ± SD]) volunteered. On day 1, they performed an incremental cardiopulmonary exercise test (CPET) on a cycle ergometer (Ergoline, Germany) till exhaustion to determine their peak aerobic power output (W_peak_) and oxygen consumption (VO_2peak_, Cosmed Quark b2, Italy). After instrumentation, the participants sat for 3 min on the ergometer before starting pedaling at 50 W (men) or 30 W (women) for 5 min after which the power was increased by 30 or 20 W/min for men and women, respectively, until voluntary exhaustion. The pedaling rate had to be kept between 70 and 90 rpm. The maximality was considered to be reached when at least two of the following criteria were met: VO_2_ plateau, respiratory quotient > 1.1, heart rate (HR) > 90% of the theoretical peak HR, or a persisting drop in pedaling rate below 60 rpm despite a strong verbal encouragement. The post–RMET CPET data were lost for one man, and the aggregate results of the remaining six men were presented.

On day 2, the participants performed a 10-km cycling TT on a road-bike mounted on a calibrated ergometer (Spin-Trainer, Technogym, Italy) in normoxia (with local altitude 380 m) or in hypoxia (AltiTrainer, Switzerland; N_2_-enriched air with an FiO_2_ of 0.135, PiO_2_ ~96 mmHg, equivalent to an altitude of ~3,500 m). On day 4, they repeated the 10-km TT in the other condition, in a balanced randomized order. The participants could see the distance covered and were instructed to complete the TTs in the shortest time possible under strong verbal encouragement all along.

The participants then completed 4 weeks of supervised RMET (5 days/week, 30 min/day eucapnic hyperpnea, in normoxia) with a partial rebreathing device (SpiroTiger, IDEAG, Switzerland) breathing at ~60% of their individual predicted maximum voluntary ventilation (FEV1 × 40 L/min) (Quanjer et al., 2012).

Upon completion of the 4 weeks of supervised RMET, the participants then repeated the three tests (day 1: incremental exercise test; next day and 48 h later: normoxic and hypoxic TTs, randomized balanced order). During the TTs, HR (E600 wrist watch, Polar, Finland), ear oximetry reading (SpO_2_, Datex Oscar 2, Finland; only in hypoxia), and W_peak_ were recorded every minute and at arrival. The participants were kept naïve concerning the specific hypothesis of the study and remained blinded to their performances and physiological parameters for all tests for the duration of the study.

The aggregate data were reported as means ± SD unless stated otherwise. The statistical analysis was performed with Prism (Version 9, Graphpad Software, San Diego, CA, USA). The normality of data distribution was verified with the Shapiro–Wilk test. For the TT performance, we used repeated measures (pre–post RMET) ANOVA with the factors woman/man and normoxia/hypoxia. For the other analyses, ANOVA or mixed-effects modeling was used, depending on any missing data. *Post-hoc* comparison of means was performed with the Sidak method. The results were considered significant when *p* < 0.05.

## Results

### Cardiopulmonary Exercise Test

There was a significant effect of sex on absolute and relative VO_2peak_ (both *p* < 0.001) and absolute and relative W_peak_ (both *p* < 0.001). The VO_2peak_ (*p* = 0.115) and W_peak_ (*p* = 0.959) values were not changed after RMET (see Table 1). The peak HR was not changed after RMET (women: 183 ± 5 and 182 ± 8; men 192 ± 7 and 193 ± 7 bpm).

**Table 1:** Results of cardiopulmonary exercise testing (CPET).

	**Pre-RMET**				**Post-RMET**			
	**Wpeak**	**Wpeak/kg**	**VO_**2**_peak**	**VO_**2**_peak/kg**	**Wpeak**	**Wpeak/kg**	**VO_**2**_peak**	**VO_**2**_peak/kg**
	**(watts)**	**(watts/kg)**	**(ml/min)**	**(ml/min/kg)**	**(watts)**	**(watts/kg)**	**(ml/min)**	**(ml/min/kg)**
Women (N = 7)	148 ± 33	2.7 ± 0.6	2,435 ± 406	44.0 ± 7.6	159 ± 26	2.9 ± 0.5	2,663 ± 361	47.9 ± 5.6
Men (N = 6)	282 ± 53	3.6 ± 0.5	4,572 ± 568	57.8 ± 2.8	271 ± 36	3.4 ± 0.4	4,578 ± 648	57.9 ± 4.4

*Mean ± SD pre vs. post values of peak power output (W_max_) and peak oxygen consumption (VO_2peak_). There were no significant effects of respiratory muscle endurance training (RMET). Men: means of N = 6; one post-RMET cardiopulmonary exercise test (CPET) measurement was lost due to technical problems*.

### Time Trial Performance

**Figure 1:**
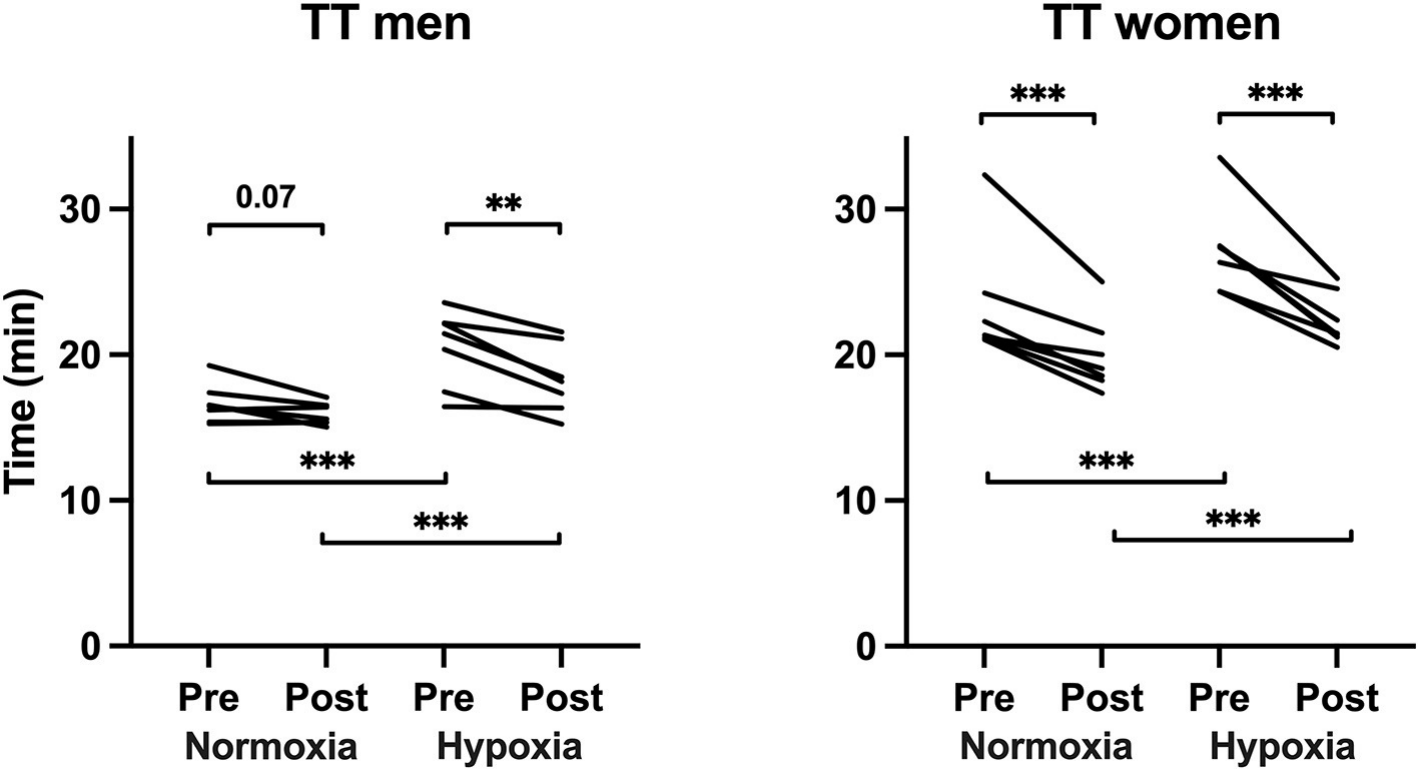
Individual times of the 10-km time trials before and after respiratory muscle endurance training (RMET). **p < 0.01 and ***p < 0.001. Pre–post RMET normoxia and hypoxia means were different between men and women: normoxia before RMET, *p* < 0.001, hypoxia before RMET, *p* < 0.001, normoxia after RMET, *p* < 0.001, and hypoxia after RMET, *p* < 0.05 (Sidak multiple comparison adjusted).

For both men and women, RMET significantly improved the TT performance more in hypoxia (by 16 ± 6%, *p* < 0.001) than in normoxia (by 7 ± 6%, *P* < 0.001) (see Figure 1). The difference between normoxic and hypoxic TT was smaller after RMET as compared with that before RMET (14% *vs*. 21%, respectively, *p* < 0.001). The effects of RMET on TT performance were greater in women (pre–post: *p* < 0.001; normoxia–hypoxia: *p* < 0.001). Women had higher average saturations during TTs in hypoxia than men (women pre-RMET, 85 ± 2, and post-RMET, 85 ± 4%; men pre-RMET, 81 ± 3, and post-RMET, 80 ± 2%, *p* < 0.001, no effect of RMET *per se*). RMET did not affect the mean nor the final levels of HR or ear oximetry reading during TT in normoxia or hypoxia (Table 2).

**Table 2:** Parameters monitored during the 10-km cycling time trials.

	**Pre-RMET**				**Post-RMET**			
	**Men**		**Women**		**Men**		**Women**	
	**Normoxia**	**Hypoxia**	**Normoxia**	**Hypoxia**	**Normoxia**	**Hypoxia**	**Normoxia**	**Hypoxia**
Wmean (watt)	240 ± 45	185 ± 34	130 ± 18^*^	105 ± 12^*^	231 ± 25	177 ± 16	150 ± 60^*^	113 ± 9^*^
Wpeak (watt)	310 ± 103	281 ± 111	157 ± 22^*^	138 ± 18^*^	267 ± 35	225 ± 42	196 ± 108^*^	138 ± 20^*^
HRmean (bpm)	169 ± 13	158 ± 11	164 ± 7	157 ± 12	171 ± 8	161 ± 10	160 ± 13	161 ± 10
HRpeak (bpm)	188 ± 14	183 ± 10	180 ± 5	177 ± 7	190 ± 7	181 ± 8	182 ± 7	177 ± 7
SpO_2_mean (%)	-	81 ± 3	-	85 ± 2^*^	-	80 ± 2	-	85 ± 4^*^
SpO_2_min (%)	-	73 ± 8	-	75 ± 8	-	75 ± 3	-	75 ± 9

*SpO_2_ was not monitored in normoxia.*

* *Significantly different from the corresponding variable for men*.

## Discussion

Based on our results, the hypotheses that (i) RMET improves performance of endurance exercise both in normoxia and in hypoxia, (ii) but more so in hypoxia, and (iii) more so in women than in men are not refuted. These findings are in agreement with earlier studies on the effects of RMET on the performance of cycling TT and contribute to the novel findings of a greater effect of RMET on the performance in 10-km TT in hypoxia than in normoxia, and an effect of sex on the extent of RMET-induced improvement of the performance in 10-km TT in both normoxia and hypoxia.

### Respiratory Muscle Endurance Training

Earlier studies remained somewhat inconclusive on the effects of RMT on high-intensity endurance-type exercise (Morgan et al., 1987; Fairbarn et al., 1991). But since the pioneering work by Boutellier and Piwko (1992), although initially received with some reserve (Sheel, 2002), numerous studies have confirmed the positive effects of RMST and RMET on respiratory muscle force and endurance and also on the performance of large muscle group–type exercises such as running or cycling (Shei, 2018). Several systematic quantitative meta-analyses now have consolidated the collective evidence in favor of RMT (Illi et al., 2012; HajGhanbari et al., 2013; Sales et al., 2016; Karsten et al., 2018; Lorca-Santiago et al., 2020).

Various devices and methods for RMT exist, such as inspiratory loaded breathing or eucapnic hyperpnea (Menzes et al., 2018). Conform the systematic review by Illi et al. (2012), we chose to use RMET consisting of 30 min/day eucapnic hyperpnea at ~60% of predicted maximum voluntary ventilation, 5 days/week, for 4 weeks, expecting improvements in 10-km TT cycling in normoxia by at least 5%. This method allows training the respiratory muscles with a hyperpnea similar to that developed during actual exercise, loading both the inspiratory and expiratory muscles, thus inducing global fatigue of the respiratory muscles (Verges et al., 2010). We found an average TT improvement of 7% in our normoxic conditions, in the expected range for healthy but not especially trained young adults (Illi et al., 2012). We deliberately chose to recruit recreationally active but not especially trained participants, since the meta-analysis by Illi et al. (2012) suggested that less-fit subjects benefit more from RMET than highly trained athletes. The latter already have more trained respiratory muscles because of their regular exposure to sustained exercise hyperpnea (Dempsey et al., 2020) and, therefore, benefit less from further RMET (Illi et al., 2012).

### Maximum Oxygen Consumption

We did not find effects of RMET on VO_2peak_ nor on W_peak_. In their meta-analysis, Illi et al. (2012) reported that out of the 22 studies that assessed VO_2peak_ before and after RMT, only two reported that VO_2peak_ was changed. Illi et al. (2012) contended that this is because the time spent exercising at high fractions of VO_2peak_, toward the end of a CPET when the respiratory muscles are most likely to fatigue, is too short to elicit sufficient fatigue of the respiratory muscle to prematurely limit the test. This reasoning is supported by their meta-regression showing that the improvement in performance after RMT was greater for longer test durations.

### Endurance Performance

Esposito et al. (2010) assessed the effects of an RMET protocol similar to ours on VO_2max_ in normoxia and hypoxia. They found that RMET improved the respiratory function but did not have any effect on VO_2max_, neither under normoxic nor under hypoxic conditions. However, they did not test if RMET improved prolonged aerobic endurance exercise in those conditions.

The effects of RMET seem to be greatest for sustained endurance efforts at intensities of 65–85% of peak aerobic power (Morgan et al., 1987; Spengler and Boutellier, 2000; Illi et al., 2012; Shei, 2018). During the normoxic TTs, our participants developed 89% of normoxic peak aerobic power on average, while in hypoxia, they were only able to maintain 69% of their normoxic peak aerobic power on average. Given that hypoxia exacerbates fatigue of the respiratory muscles for matched ventilatory patterns (Verges et al., 2010) and hypoxia leads to an increase in ventilation, these intensities were likely high enough to elicit fatigue of the respiratory muscles during the TTs. The increased TT performance after RMET thus probably was the result of an improved training status and fatigue resistance of the respiratory muscles of our participants (Segizbaeva et al., 2014).

Given that exercise in hypoxia exacerbates fatigue of the respiratory muscles (Verges et al., 2010), we surmised that the effect of RMET would be greater in hypoxia as compared with that in normoxia. In agreement with our study, the increase of average performance in normoxia after RMET was 7%, while in hypoxia it reached 16%. While numerous studies have reported positive effects of RMT on endurance performance in normoxia (Illi et al., 2012; HajGhanbari et al., 2013; Sales et al., 2016; Karsten et al., 2018; Shei, 2018; Lorca-Santiago et al., 2020), only few studies have looked at the effects of RMT in normoxia on performance in hypoxia.

Hursh et al. (2019) reported that inspiratory muscle training (daily repeated full inspirations against 80% of maximal inspiratory pressure [MIP] for 6 weeks) in well-trained cyclists increased VE and VO_2_ during a 20-km cycling TT test, improving it by 1.4% while breathing 16% O_2_ (equivalent to an altitude of 2,500 m). We tentatively explained our greater effect from our RMET protocol, the lower FiO_2_, and the less trained participants. Salazar-Martínez et al. (2017) found that 6 weeks of inspiratory pressure-threshold RMST improved ventilatory efficiency in both normoxia and hypoxia (VE/VCO_2slope_) from the beginning of exercise until the second ventilatory threshold. The performance of cycling TT (10-min all out) was improved in both normoxia and hypoxia after RMST, and TT performance was correlated with the slope of oxygen uptake efficiency but not with VE/VCO_2slope_. Lomax et al. (2017) found that ventilatory efficiency, estimated by the ratio of peripheral capillary oxygen saturation (SpO_2_) to VE (SpO_2_/VE), was improved after hypoxic inspiratory RMT but not after normoxic inspiratory RMT. Held and Pendergast (2014), in conditions of hyperbaria, also reported an increased ventilatory efficiency following RMST, possibly linked to altered pulmonary mechanics and breathing patterns.

Keramidas et al. (2010) found, in healthy physically active but untrained men, that 4 weeks of daily cycling training at 50% of aerobic power combined with an RMET protocol similar to ours improved time to exhaustion in normoxia at 80% of peak aerobic power more compared with the group that only performed cycling training and no RMET, but not in hypoxia. They explained this somewhat paradoxical finding mentioning the greater intensity of relative exercise used in their hypoxic constant load test to exhaustion. Their design used the same absolute workload equivalent to 80% of normoxic pre-RMET peak aerobic power, leading to very high relative intensities in hypoxia (92% of pre-RMET peak aerobic power), too high probably for a discernable effect of RMET.

Katayama et al. (2019), in male intercollegiate competitive runners, compared 6 weeks of eucapnic hyperpnea RMET similar to ours performed either in normoxia or in hypoxia (first at 90% and then at 80% SaO_2_) for 6 weeks. Time to exhaustion at 95% of VO_2peak_ in normoxia was increased after RMET to a similar extent in both groups (9–12%). No performance test was conducted in hypoxia. However, the response of exercise blood pressure was attenuated after RMET similarly in both groups, suggesting a decrease of the metaboreflex of the respiratory muscles.

Hypoxia comprises aerobic exercise capacity, and the respiratory system likely plays a bigger role in limiting performance at high altitude than at low altitude (Kayser, 2005). A compensatory hyperventilation aiming to offset the effect of a reduction in inspired oxygen tension leads to a greater load on the respiratory muscles for a given metabolic rate. The combination of more respiratory work and hypoxemia together causes early fatigue of the respiratory muscles, contributing to the limitation of endurance-type performance at high altitude (Helfer et al., 2016). RMET may thus be a valuable strategy to prevent or delay these mechanisms. Downey et al. (2007) tested this hypothesis with an inspiratory load RMST protocol but found no effect on time to exhaustion at 85% of peak normoxic aerobic power, either in normoxia or in hypoxia (14% O_2_, equivalent of 3,200 m). The absence of effects in their study may be explained by the use of an inspiratory muscle training protocol, while expiratory muscles also likely play a role (Verges et al., 2010), a respiratory pattern that differs from that experienced during hyperpnea, and the very high intensity of their test in hypoxia (85% peak normoxic aerobic power) leading to a time to exhaustion 50% less than in normoxia. By contrast, Helfer et al. (2016) used an RMET protocol similar to ours, which produces a breathing pattern resembling that of exercise-induced hyperpnea, and found that it improved exercise time to exhaustion at 75% of peak aerobic power in hypoxia by 44%. Our finding of a relatively greater effect of RMET on the performance in 10-km cycling TT in hypoxia as compared with that in normoxia suggests that RMET may be of a particular interest for those who want to prepare for endurance-type efforts at high altitudes, such as trekkers, climbers, trail-runners, and cyclists.

### Sex Difference

Women and men differ with regard to exercise physiology (Ansdell et al., 2020) and, in specific, also with regard to respiratory physiology (Sheel et al., 2016). Women are more susceptible to limitations of the respiratory system during exercise than men (Molgat-Seon et al., 2018; Dempsey et al., 2020), and this difference is likely to be exacerbated in hypoxia. We found that the effect of RMET on the performance in 10-km cycling TT was greater in women than in men. Why would women have profited more from RMET in hypoxia in our study as compared with men? Geary et al. (2019) found that, in normoxia, matching men and women for absolute diaphragmatic work resulted in an equal degree of diaphragm fatigue, despite women performing significantly greater work relative to body mass. Archiza et al. (2020) compared diaphragm muscular strength-matched healthy women and men upon 5 min of inspiratory loading at equal absolute muscle work both in normoxia and in hypoxia. While in normoxia, diaphragm fatigue was similar in both men and women, when acutely exposed to severe hypoxia (SpO_2_ ~80% and CaO_2_ ~16 ml/dl), the diaphragm fatigue was worsened only in women, suggesting that contrary to normoxic condition, the healthy female diaphragm would be more susceptible to fatigue under hypoxic condition. Perhaps, the effect of RMET in women was therefore proportionally greater in hypoxia than normoxia in comparison with men in our study.

The study of Salazar-Martínez et al. (2017) included 16 participants of which seven were women. Effects of sex were not a primary outcome of the study, but the authors stated that there were no significant differences between men and women for the change in the 10-min all-out TT in hypoxia and normoxia. At least two explanations may be advanced for their negative findings. First, they used an inspiratory loading protocol and not a more ecological valid RMET protocol such as ours, and second, their endurance test was probably performed at a too high intensity to reveal any effects of such training on the endurance of the respiratory muscles. Similarly, Guenette et al. (2006) found no difference between men and women using an inspiratory loading training protocol (5 days/week, two sets of 30 inspirations against 50% MIP) and a different endurance test, (time to exhaustion at 80% peak aerobic power). We contended that such designs do not adequately allow testing the hypothesis that global RMET, including both inspiratory and expiratory muscles and using breathing patterns that resemble those experienced during exercise-induced hyperpnea, can improve the performance of prolonged aerobic endurance.

## Limitations

Our results should be interpreted taking into account several important methodological limitations. First, placebo as well as nocebo effects can occur, also in exercise studies (Hurst et al., 2019). The difficulty is to design adequate placebo or sham intervention arms, which for the eucapnic hyperpnea that we used as *verum* is complicated. Since in their quantitative meta-analysis, only 43% of the included RMT studies included a sham-training group to account for a possible placebo effect of RMT, Illi et al. (2012) could specifically look if it made any difference to have a sham or placebo group in RMT studies. Their analysis suggested that the presence and type of such a control group did not make a difference. Also, our finding of unchanged VO_2peak_ after RMET corroborates this contention. Overall, we deemed our choice to use a pre–post hypoxia–normoxia cross-over design without a sham condition to be a reasonable trade-off but cannot fully exclude that placebo and/or training effects could have influenced our results. Second, we did not specifically quantify the effects of RMET on respiratory muscle force and performance with maximum inspiratory pressure or respiratory endurance test to see if RMET had greater effects in women as compared with men. Third, even though our participants were young active people who were not engaged in structured athletic training, the men were somewhat more physically active as compared with the women and, therefore, relatively better trained, in part reflected in their respective VO_2peak_ values, which may have led to a different effect of the RMET in the two groups. Fourth, our sample size was rather small, and our study needs replication beyond our pilot approach. Fifth, we did not quantify locomotor nor respiratory muscle fatigue after the TT to see if it was changed, nor did we quantify any other metaboreflex-caused cardiovascular changes after RMET. Sixth, for technical reasons, we could not collect respiratory data during the TTs and, therefore, could not compare breathing patterns during the TTs, before and after RMET. Seventh, we chose a 10-km cycling TT for assessing the effects of RMT. The relatively greater effects of RMET on the TT performance in hypoxia may in part also have been due to the fact that the duration of exercise for the same distance was greater in hypoxia than in normoxia. Also, since we did not implement TT familiarization sessions, we could not exclude that some learning effects may have influenced our results. Finally, we did not collect any data allowing to look into the potential mechanistic explanations for our findings. Follow-up studies are needed to look into the possible mechanistic explanations underlying the differing effects of RMET on endurance performance in women compared with men.

## Conclusion

Women are more prone to limitation by the respiratory system of endurance-type exercise performance. Apart from limitation of expiratory flow, fatiguability of the respiratory muscles may also play a role. We found that eucapnic hyperpnea training 5 × 30 min/week for 4 weeks significantly improved the performance in the 10-km TT, more in women than in men, and more so in hypoxia than in normoxia. This greater effect of RMT on TT performance in hypoxia than in normoxia is compatible with the contention of a more pronounced performance-limiting role for the respiratory system during endurance exercise in hypoxia compared with normoxia, and more so in women whose respiratory system is undersized compared with that of men. Due to the limitations of our pilot study design, these results need to be confirmed in trained athletes and complemented with additional exploration of explanatory mechanisms. These would include quantifying the training-induced respiratory muscle strength and endurance, and any attenuation of the respiratory muscle metaboreflex and of the decreased rating of perceived breathlessness or rating of perceived exertion (Shei, 2018). Our results suggest a further potential for RMET for improving the endurance performance, specifically for performance at high altitudes and especially for women.

## Data Availability Statement

The raw data supporting the conclusions of this article will be made available by the authors, without undue reservation.

## Ethics Statement

The studies involving human participants were reviewed and approved by Comité d'éthique de la recherche du Canton de Genève. The participants provided their written informed consent to participate in this study.

## Author Contributions

JC and GG collected the experimental data. All authors analyzed the data. BK wrote the first draft of the manuscript. All authors contributed to the design of the protocol, final article, and approved the submitted version.

## Conflict of Interest

The authors declare that the research was conducted in the absence of any commercial or financial relationships that could be construed as a potential conflict of interest.

## Publisher's Note

All claims expressed in this article are solely those of the authors and do not necessarily represent those of their affiliated organizations, or those of the publisher, the editors and the reviewers. Any product that may be evaluated in this article, or claim that may be made by its manufacturer, is not guaranteed or endorsed by the publisher.
